# MAGUS+eHMMs: improved multiple sequence alignment accuracy for fragmentary sequences

**DOI:** 10.1093/bioinformatics/btab788

**Published:** 2021-11-17

**Authors:** Chengze Shen, Paul Zaharias, Tandy Warnow

**Affiliations:** Department of Computer Science, University of Illinois Urbana-Champaign, Urbana, IL 61801, USA; Department of Computer Science, University of Illinois Urbana-Champaign, Urbana, IL 61801, USA; Department of Computer Science, University of Illinois Urbana-Champaign, Urbana, IL 61801, USA

## Abstract

**Summary:**

Multiple sequence alignment is an initial step in many bioinformatics pipelines, including phylogeny estimation, protein structure prediction and taxonomic identification of reads produced in amplicon or metagenomic datasets, etc. Yet, alignment estimation is challenging on datasets that exhibit substantial sequence length heterogeneity, and especially when the datasets have fragmentary sequences as a result of including reads or contigs generated by next-generation sequencing technologies. Here, we examine techniques that have been developed to improve alignment estimation when datasets contain substantial numbers of fragmentary sequences. We find that MAGUS, a recently developed MSA method, is fairly robust to fragmentary sequences under many conditions, and that using a two-stage approach where MAGUS is used to align selected ‘backbone sequences’ and the remaining sequences are added into the alignment using ensembles of Hidden Markov Models further improves alignment accuracy. The combination of MAGUS with the ensemble of eHMMs (i.e. MAGUS+eHMMs) clearly improves on UPP, the previous leading method for aligning datasets with high levels of fragmentation.

**Availability and implementation:**

UPP is available on https://github.com/smirarab/sepp, and MAGUS is available on https://github.com/vlasmirnov/MAGUS. MAGUS+eHMMs can be performed by running MAGUS to obtain the backbone alignment, and then using the backbone alignment as an input to UPP.

**Supplementary information:**

Supplementary data are available at *Bioinformatics* online.

## 1 Introduction

Multiple sequence alignment (MSA) is a critical precursor for many downstream analyses, such as gene and species tree estimation (Heled and Drummond, 2010; Stamatakis, 2014), protein family classification (Nguyen *et al.*, 2016) and phylogenetic placement (Matsen *et al.*, 2010). Because of the broad impact of MSA estimation, the development of new alignment methods continues to be of interest in the community (e.g. see Katoh, 2021 for a recent book on MSA estimation).

One of the challenges that arise in MSA estimation is sequence length heterogeneity, which occurs naturally in many datasets due to evolutionary processes that include large insertions or deletions (jointly called ‘indels’), and also when the input sequence datasets include some incompletely assembled sequences (e.g. contigs or reads). Datasets of interest may contain some full-length sequences as well as fragmentary sequences, for example when performing taxonomic identification of reads in microbiome samples as in TIPP (Nguyen *et al.*, 2014; Shah *et al.*, 2021).

To produce MSAs on datasets that contain both full-length and fragmentary sequences, techniques for adding fragmentary sequences into alignments of (generally) full-length sequences have been developed, including MAFFT –addfragments (Katoh and Frith, 2012) and techniques based on ensembles of profile HMMs (Mirarab *et al.*, 2012; Nguyen *et al.*, 2015). These methods, when provided with good alignments on the full-length sequences, have been shown to provide better accuracy than alignment methods that do not explicitly take fragmentary sequences into account. In particular, UPP (Nguyen *et al.*, 2015), which constructs a ‘backbone alignment’ on a sample of the full-length sequences using PASTA (Mirarab *et al.*, 2015) and then adds the remaining sequences into the backbone alignment using an ensemble of profile HMMs (i.e. eHMMs) technique, was shown to provide very good accuracy and scalability to large and ultra-large datasets (up to 1 000 000 sequences), even in the presence of high levels of fragmentary sequences.

Here, we address the possibility of improving alignment accuracy compared with UPP. The main change we explore is the use of MAGUS (Smirnov and Warnow, 2020a), a new alignment method that has been shown to be generally more accurate than PASTA, for the backbone alignment. We also explore the use of techniques from MAFFT for adding sequences into the backbone alignment. We use both simulated and biological datasets to evaluate the alignment methods we explore in this study. Our results show that the best method in terms of alignment accuracy is by using MAGUS for the backbone alignment and then an eHMM for adding in the remaining sequences. This new approach, which we call MAGUS+eHMMs, matches or improves on both MAGUS and UPP, particularly when aligning datasets that evolved under high rates of evolution and that have large fractions of fragmentary sequences.

## 2 Experimental study design

### 2.1 Overview

We performed three experiments to design and evaluate methods for MSA estimation on datasets that have fragmentary sequences. In Experiment 1, we explore the design space for computing the backbone alignment and adding sequences to the backbone alignment. We show that the best variant is to use MAGUS for backbone alignment and eHMMs for adding the remaining sequences, and denote it as MAGUS+eHMMs. In Experiment 2, we compare MAGUS to MAGUS+eHMMs and show that MAGUS+eHMMs improves or matches MAGUS alignment performance. In Experiment 3, we evaluate MAGUS+eHMMs in comparison to methods that have been shown to have high alignment accuracy. We again show that it is the overall best performing method.

Due to space limitations, commands needed to reproduce the experiment, as well as we also additional figures and tables, are in Supplementary Materials available online at https://tandy.cs.illinois.edu/magus-ehmms-suppl.pdf.

### 2.2 Methods

In this study, we evaluate two-stage methods and other multiple sequence alignment tools (i.e. MAFFT, PASTA, MAGUS and MUSCLE) on datasets with and without fragmentation. Other methods such as Clustal-Omega (Sievers *et al.*, 2011) are not included, because they are shown to be less accurate on the selected datasets (Mirarab *et al.*, 2015; Nguyen *et al.*, 2015).

**MAFFT** (Katoh and Standley, 2013) is a well-known alignment method that has performed very well in many studies evaluating alignment methods (Edgar and Batzoglou, 2006; Nuin *et al.*, 2006; Sievers *et al.*, 2011). Some of its variants (e.g. the L-INS-i and G-INS-i options that use iterative refinement) have particularly strong alignment accuracy. MAFFT also provides options to add full-length or fragmentary sequences to an existing alignment (e.g. the ‘–addfragments’ option). We run MAFFT in two ways for aligning datasets: the default setting (which is faster but less accurate) and the L-INS-i setting (which is slower but generally more accurate). We also run MAFFT to add sequences into backbone alignments using its options described above.

**MUSCLE** (Edgar, 2004) is another well-known multiple sequence alignment method. We run MUSCLE in default mode on datasets with at most 3000 sequences and with two iterations on larger datasets.

**PASTA** (Mirarab *et al.*, 2015) is a method that combines iteration with divide-and-conquer to co-estimate alignments and trees, where each iteration begins by dividing the dataset into subsets using the tree from the previous iteration, aligning the subsets using a selected method (e.g. MAFFT L-INS-i), merging the alignments using OPAL (Wheeler and Kececioglu, 2007) or Muscle (Edgar, 2004) along with transitivity, and then computing a tree on the merged alignment. PASTA has been shown to have better alignment accuracy than its predecessors SATé (Liu *et al.*, 2009) and SATé-II (Liu *et al.*, 2012). We run PASTA in its default mode.

**MAGUS** (Smirnov and Warnow, 2020a) is a recently developed alignment method that improves on PASTA for alignment accuracy. By design, it is similar to PASTA (but with specific changes that impact its accuracy). It begins with an initial alignment and tree, decomposes the tree into a user-specified number of subsets, and aligns the subsets using MAFFT L-INS-i. Then it differs from PASTA: it combines the subset alignments using a novel approach where it first computes additional estimated alignments, uses these to define weights on pairs of columns from different alignments, and then merges these disjoint subset alignments using the Graph Clustering Merger (GCM) method (which is essentially the Markov Clustering technique (Van Dongen, 2000a,b) with some extensions to ensure that a legal alignment is created). MAGUS has been shown to have better alignment accuracy than PASTA and other methods, but has only been tested on full-length datasets (Smirnov and Warnow, 2020a). The current version of MAGUS in GitHub defaults to a recursive approach on subset alignments if they have more than 200 sequences; however, Smirnov (2021) notes that ‘recursion does not improve accuracy’ and ‘should be avoided if possible, and only engaged when the dataset becomes too large for the subsets to be reasonably aligned with the base method’. Therefore, we use MAGUS without recursion.

**UPP** (Nguyen *et al.*, 2015) is a divide-and-conquer alignment method that operates in steps described in the Introduction. UPP improves on PASTA and other methods in terms of alignment accuracy when the datasets have fragmentary sequences (Nguyen *et al.*, 2015). We run UPP in its default mode.

In general, we will describe UPP as a *two-stage* method in which the first stage selects and aligns the backbone sequences and the second stage adds the remaining sequences into the backbone alignment. In contrast, the other methods (MAFFT, PASTA and MAGUS), as well as most standard alignment methods, are not based on this type of two-stage approach and considered as *one-stage* methods in the context of this study.

### 2.3 Datasets

We explore alignment methods on a collection of biological and simulated datasets, some with introduced fragmentation (as described below). The biological datasets all have reference alignments based on structure, and the simulated datasets have true alignments. Nearly, all the datasets used in this study are from prior studies, and available in public databases (see Supplementary Materials); the remaining datasets are available in the Illinois Data Bank (see availability statement). The empirical statistics for these datasets are provided in Tables  1 and 2. These datasets vary in number of sequences (from 278 to nearly 27 643), degree of heterogeneity (as indicated by the average and maximum p-distances) and the percentage of the reference or true alignment is occupied by gaps. All sequence alignments have sequences of approximately the same pre-alignment length (averaging 1000–2000 nucleotides).

**Table 1.** Empirical dataset properties for the datasets with introduced fragmentation

Dataset	No. of Seqs.	p-distance		% gaps	Avg. seq. length	Avg. alignment length	No. of frag. seqs (L/H)	Avg. frag. length (L/H)
		Avg.	Max					
Simulated datasets								
10K(10)	10 000	0.411	0.629	89.6	1551	17 955	–/5000	–/387
1000M1(19)	1000	0.694	0.781	74.3	1011	3960	250/500	505/251
1000M2(20)	1000	0.684	0.775	74.2	1014	3972	250/500	505/254
Biological datasets								
16S.M(1)	901	0.359	0.887	78.1	1035	4722	225/450	473/240
23S.M(1)	278	0.377	0.703	83.7	1746	10 738	69/139	785/392
16S.3(1)	6323	0.315	0.833	82.1	1557	8716	–/3161	–/373
16S.T(1)	7350	0.345	0.901	87.4	1492	11 856	–/3675	–/368
16S.B.ALL(1)	27 643	0.210	0.769	79.9	1372	6857	–/13 821	–/363

*Note*: The empirical statistics are computed per-replicate (number of replicates is marked next to dataset names). The p-distance between two aligned sequences is the fraction of the sites in which they have different nucleotides. Percent gapped is the percentage of the alignment matrix occupied by dashes. 1000M1 has replicate 16 removed due to being identified as an outlier in Smirnov and Warnow (2020b). Statistics regarding fragments are computed after introducing fragments to the datasets, while the rest are computed prior to fragmentation. (L/H) in the last two columns indicates high or low level of fragmentation.

**Table 2.** Empirical dataset properties for 14 CRW datasets without introduced fragmentation

Dataset	# Seqs.	p-distance		% gaps	Avg. seq. length	Align length
		Avg.	Max			
5S.E	2774	0.305	1.000	87.8	96	793
5S.3	5507	0.418	1.000	74.5	105	414
5S.T	5751	0.425	1.000	75.6	106	436
16S.A	594	0.185	0.673	85.8	1103	7774
16S.B.ALL	27 643	0.210	0.769	80.0	1372	6857
16S.C	320	0.157	1.000	86.9	1017	7774
16S.M	805	0.359	0.768	78.8	1042	4931
16S.3	6323	0.315	0.833	82.1	1557	8716
16S.T	7350	0.345	0.901	87.4	1492	11 856
23S.A	214	0.293	0.667	53.6	1851	3991
23S.C	374	0.143	0.750	64.7	2086	5916
23S.E	105	0.291	0.513	61.5	3635	9436
23S.M	254	0.380	0.695	84.0	1761	10 999
23S.3	451	0.337	0.544	78.3	3091	14 244

*Note*: The p-distance between two aligned sequences is the fraction of the sites in which they have different nucleotides. Percent gapped is the percentage of the alignment matrix occupied by dashes.

*Fragmentary versions of datasets.* We explore two techniques for making fragmentary versions of our datasets. The script to generate an alignment with fragments is available at https://git.io/JOGO1.

- *High fragmentary (HF)* means that 50% of the sequences are made into fragments. The fragment size is sampled from a normal distribution with mean *M* (where *M* corresponds to 25% of the original median sequence length) and a standard deviation of 60.
- *Low fragmentary (LF)* is similar to HF except that only 25% of the sequences are made into fragments and *M* corresponds to 50% of the original median sequence length.

*Simulated datasets.* We use ROSE and RNASim datasets, each from prior studies. The ROSE datasets are simulated DNA sequence datasets generated using the ROSE simulator (Stoye *et al.*, 1998) and previously studied by Liu *et al.* (2009). These sequences evolve under the GTRGAMMA model with various indel rates. Each condition has 20 replicates and each replicate contains 1000 sequences with ∼ 1000 nucleotides. We explore fragmentary versions of the 1000M1 and 1000M2 conditions (also studied by Smirnov and Warnow, 2020b), which have high and moderately high rates of evolution, respectively, and medium indel lengths. The RNASim (Mirarab *et al.*, 2015) datasets evolve under a complex evolutionary process that reflects selective pressures needed to conserve rRNA structure; hence, this simulation condition is more complex than the standard GTR+indel simulations (such as for the ROSE datasets). The original RNASim dataset contains 1 000 000 sequences, but we explore subsets with 10 000 sequences.

*Biological datasets.* We use the Comparative RNA Website (CRW) datasets (Cannone *et al.*, 2002), which are alignments of RNA sequences based on structure. We explore 5S.E, 5S.3, 5S.T, 16S.A, 16S.C, 16S.M, 23S.A, 23S.C, 23S.E, 23S.M and 23S.3 datasets. We then remove any sequence that contains any ambiguity codes or that is entirely gapped. In addition, we examine the large 16S.3, 16S.T and 16S.B.ALL datasets, which were originally from the CRW and then used by Liu *et al.* (2009), Mirarab *et al.* (2015).

*Training versus Testing data.* We use the high-fragmentation version of the RNASim 10K dataset as our training data for Experiment 1 and the remaining datasets for our other experiments.

### 2.4 Criteria

Our main criterion is alignment error (false-positive and false-negative rates, as well as their average), computed by comparing the estimated alignment to the reference alignment. Each alignment is represented with its set of pairwise homologies, which allows for the calculation of false positive homologies (pairs of nucleotides aligned in the estimated alignment that are not aligned in the reference alignment) and false negative homologies (pairs of nucleotides aligned in the reference alignment that are not aligned in the estimated alignment). The SPFN (sum-of-pairs false negative) rate is the fraction of the true pairwise homologies that the estimated alignment fails to recover, and the SPFP (sum-of-pairs false positive) rate is the fraction of the pairwise homologies in the estimated alignment that are not in the reference alignment. We compute SPFN and SPFP using FastSP (Mirarab and Warnow, 2011). We also report the average of these two-error metrics. We evaluate wall clock running time. However, due to the heterogeneous nature of the University of Illinois Campus Cluster and the Blue Water server, it is not possible to make clear inferences from these runtime comparisons.

### 2.5 Experiments

We performed three experiments. In Experiment 1, we explore variants on the two-stage algorithmic strategy for aligning datasets that contain both full-length and fragmentary sequences. We evaluate their performance on the RNASim 10K-HF dataset with respect to both alignment error (measured using SPFN and SPFP) and wall clock running time. MAGUS+eHMMs is the most accurate method (with an accuracy advantage when using all full-length sequences for its backbone alignment and a running time advantage when using only 1000 sequences for the backbone). For the remaining experiments, we fix the backbone alignment size in MAGUS+eHMMs to only 1000 sequences. In Experiment 2, we compare MAGUS+eHMMs to MAGUS on the full range of testing datasets, and demonstrate the superiority of MAGUS+eHMMs. Finally, in Experiment 3, we compare MAGUS+eHMMs to two versions of MAFFT (default and L-INS-i), PASTA and UPP on the full range of testing datasets.

Here, we provide additional details regarding Experiment 1. As described earlier, the basic strategy samples a desired number of sequences considered to be full-length, computes a ‘backbone alignment’ on these sequences using a preferred method, and then adds the remaining sequences into the backbone alignment using a selected strategy. We explore variants for each step, as follows:

- Backbone size: 1000 (‘1000bb’) or all (‘allbb’) full-length sequences.
- Backbone alignment method: MAGUS or PASTA.
- Technique to add sequences into the backbone alignment: ‘eHMMs’ as used in UPP or MAFFT options (–add, –addfull and –addfragments, further details below).

In our experiments on datasets with introduced fragmentation, we know which sequences are fragmentary and which are full-length, and we provide this information to the two-stage methods so that the backbone alignment is computed only on the full-length sequences. In the experiments on datasets without introduced fragmentation, we use the default technique from UPP to define the full-length sequences (from which we then select the backbone sequences): all sequences within 25% of the median sequence length.

To use the eHMMs approach, we need to build eHMMs on the backbone alignment, which requires the construction of a phylogenetic tree on the backbone alignment. This is automatically performed within UPP, but when we run MAGUS+eHMMs, which utilizes the UPP code library to operate, we need to provide UPP with the backbone alignment and a tree on the backbone alignment. We perform this by computing a maximum likelihood tree on the backbone alignment using FastTree 2 (Price *et al.*, 2010).

We performed preliminary analyses for how to use MAFFT to add sequences into the backbone alignment. We first tried using the L-INS-i version of MAFFT –addfragments (which we would expect to be the most accurate setting), but this option encountered out-of-memory issues on the RNASim 10K-HF datasets (see Supplementary Materials); hence, we used the default setting for MAFFT –addfragments. We considered several versions of this technique:

1. Use ‘–addfragments’ for all missing sequences
2. Use ‘–add’ to add the missing full-length sequences, then ‘–addfragments’ to add fragments
3. Same as 2 except using ‘–addfull’ for the missing full-length sequences.

The first version is denoted by ‘MAFFT(frag)’ (i.e. only using ‘–addfragments’ to add the remaining sequences) and the other versions are denoted by ‘MAFFT(frag, add)’ (i.e. using ‘–add’ for full-length sequences and ‘–addfragments’ for fragmentary sequences) or ‘MAFFT(frag, addfull)’, depending on whether ‘–add’ or ‘–addfull’ is used.

Given the details provided, we denote different variants of the two-stage algorithmic strategy in the form of ‘[backbone alignment method]+[strategy to add the remaining sequences]+[number of sequences in the backbone]’. For example, ‘MAGUS+eHMMs + 1000bb’ refers to using 1000 full-length sequences and MAGUS to construct the backbone alignment, and using eHMMs to align the remaining sequences to the backbone alignment (and thus, to form the final alignment). ‘PASTA+MAFFT(frag)+allbb’ refers to using all full-length sequences and PASTA to construct the backbone alignment, and using MAFFT ‘–addfragments’ option to align the remaining sequences to the backbone alignment.

## 3 Results

We show results for Experiments 1–3; however, MAFFT(L-INS-i) did not complete on some datasets. We show results on individual datasets where MAFFT(L-INS-i) completed in Supplementary Materials.

### 3.1 Experiment 1 results

*Designing methods that use MAGUS for the backbone.*  Supplementary Figure S1 shows the alignment error and runtime for the variants using MAGUS as the backbone alignment method (see Supplementary Fig. S4 for SPFN and SPFP). MAGUS+eHMMs+allbb has the lowest error of all methods, but it is followed very closely by MAGUS+MAFFT(frag)+allbb and then closely by MAGUS+eHMMs + 1000bb. The other three methods have much higher error rates. Between these three methods with high accuracy, only MAGUS+eHMMs + 1000bb is reasonably fast; the other two are much slower. Furthermore, MAGUS+eHMMs + 1000bb is the fastest of the six tested pipelines. Thus, MAGUS+eHMMs+allbb is the best choice if alignment accuracy is the primary objective, but when the runtime is also considered, then MAGUS+eHMMs + 1000bb is the best choice. We use MAGUS+eHMMs + 1000bb in the subsequent experiments, and refer to it henceforth as ‘MAGUS+eHMMs’.

*Backbone tree using initial versus backbone alignment.* Recall that MAGUS first computes an initial alignment that is not designed to provide high accuracy on the backbone sequences, then estimates an initial tree using FastTree 2, and finally produces a new (improved) alignment (i.e. backbone alignment). Here, we evaluate the impact of changing how we compute the backbone tree: using the initial tree (which is computed on the initial alignment) instead using of FastTree 2 to compute a tree on the backbone alignment. This evaluation (Supplementary Fig. S8) shows that there is no noticeable difference in alignment error or runtime, while the tree estimated on the backbone alignment has slightly lower tree error.

*Running MAFFT(L-INS-i) ‘–addfragments’ with reduced memory requirement.* Since MAFFT(L-INS-i) has high memory requirements, we explored the use of the ‘–weighti 0’ option to reduce these requirements (denoted as ‘MAGUS+MAFFT(linsi-frag)+1000bb’). This approach produces less accurate alignments than MAGUS+eHMMs (on the same 1000-sequence MAGUS backbone alignments) and the guide tree it produces has much higher tree error than the MAGUS backbone tree (Supplementary Fig. S9).

*Designing methods that use PASTA for the backbone*. There is a clear best variant when using PASTA for the backbone alignment, whether with respect to running time or accuracy: PASTA+eHMMs + 1000bb (Supplementary Fig. S2, see Supplementary Fig. S3 for SPFN and SPFP). We refer to this variant as ‘PASTA+eHMMs’ and use it in the subsequent experiments. Note that, PASTA+eHMMs is identical to default UPP.

### 3.2 Experiment 2 results

**Fig. 1.**
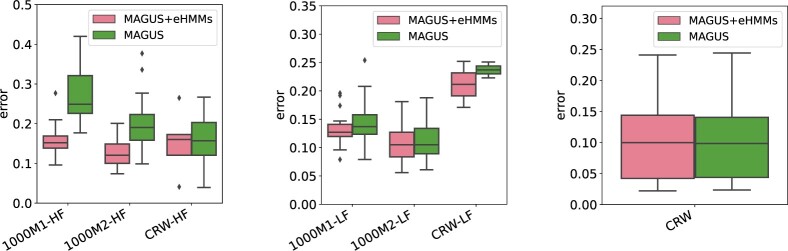
Experiment 2: Average alignment error in high fragmentation, low fragmentation, and without introduced fragmentation for MAGUS and MAGUS+eHMMs, from left to right. ‘CRW’ refers to datasets originated from Comparative Ribosomal Website (Cannone *et al*., 2002). ‘CRW-HF’ contains the high-fragmentation version of 16S.M, 23S.M (smaller datasets), 16S.3, 16S.T and 16S.B.ALL (larger datasets), ‘CRW-LF’ contains the low-fragmentation version of 16S.M and 23S.M, and ‘CRW’ contains the 14 CRW datasets without introduced fragmentation. For both high- and low-fragmentation conditions, we examine ROSE 1000M1, 1000M2 and CRW 16S.M, 23S.M datasets. For the high-fragmentation condition, we examine three additional large CRW datasets (16S.3, 16S.T and 16S.B.ALL with high fragmentation). For individual CRW dataset results with and without introduced fragmentation, see Supplementary Tables S1 and S3

The comparison between MAGUS+eHMMs and MAGUS (Fig.  1) shows that MAGUS+eHMMs matches or improves on MAGUS under all tested conditions. MAGUS+eHMMs is much more accurate than MAGUS on 1000M1 and 1000M2 with high fragmentation, somewhat more accurate on 1000M1 and 1000M2 with low fragmentation, and then has similar accuracy under the remaining conditions (i.e. on the CRW datasets, with or without fragmentation). 

### 3.3 Experiment 3 results

We compare MAGUS+eHMMs to PASTA+eHMMs (i.e. default UPP), PASTA, MAFFT(L-INS-i) and MUSCLE in terms of alignment accuracy. On some model conditions, MAFFT(L-INS-i) results are not shown due to out-of-memory issues. We explored alternative ways to run MAFFT(L-INS-i) to address these memory issues. These approaches did enable MAFFT(L-INS-i) to complete, but produced alignments that were clearly worse than alternative approaches (e.g. MAGUS+eHMMs) (Supplementary Fig. S9). Full results can be found in Supplementary Tables S1, S3 and S4.

**Fig. 2.**
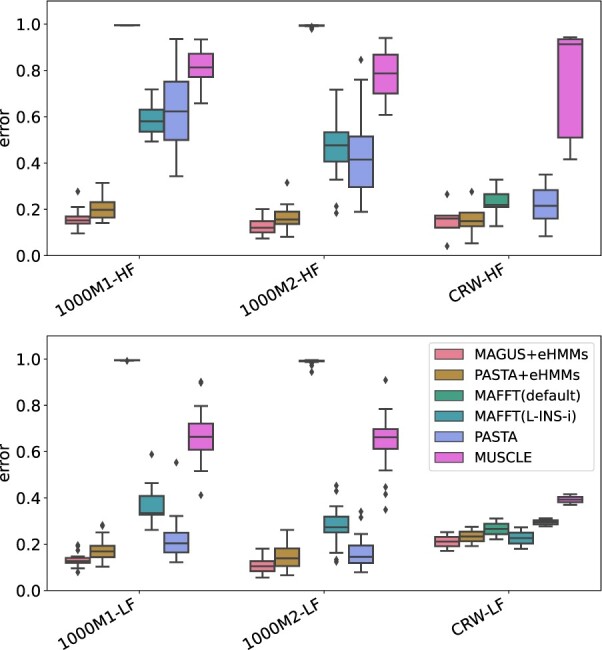
Experiment 3: Average alignment error on high-fragmentation (top) and low-fragmentation (bottom) conditions. ‘CRW-HF’ and ‘CRW-LF’ refer to the same datasets, as described in Figure  1. For ‘CRW-HF’, MAFFT(L-INS-i) is not shown as it failed to complete on one or more datasets due to out-of-memory issues. See Supplementary Table S1 for individual CRW dataset results with fragmentation

Figure  2 shows the alignment error of each method on datasets with high- and low-fragmentation conditions. MAFFT(L-INS-i) is not shown for ‘CRW-HF’ as it failed to complete on one or more datasets due to out-of-memory issues (see Section 3.4). One immediate observation is that methods are more clearly differentiated on the high-fragmentation conditions, and so group into two sets: the two better performing methods (MAGUS+eHMMs and PASTA+eHMMs, with MAGUS+eHMMs clearly better) and the four less accurate methods. On the low-fragmentation conditions, the differences between methods decrease, but MAGUS+eHMMs and PASTA+eHMMs still remain the best two, and MAGUS+eHMMs still clearly the best performing.

**Fig. 3.**
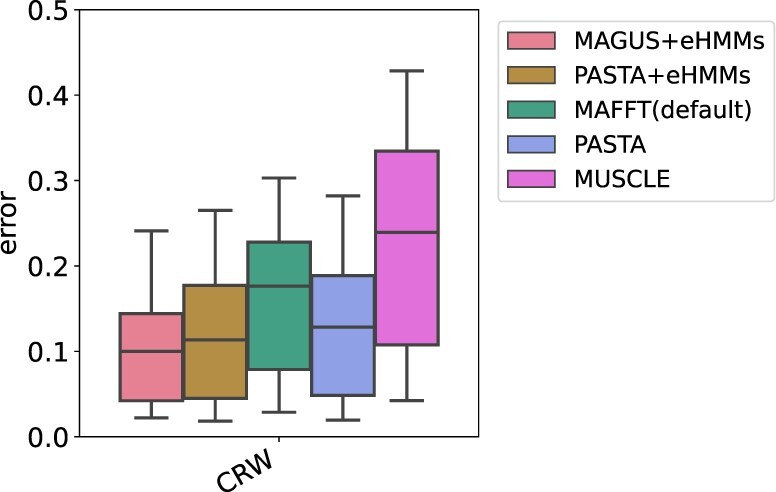
Experiment 3: Average alignment error on the 14 CRW datasets without introduced fragmentation. MAFFT(L-INS-i) could not complete on 16S.3, 16S.T and 16S.B.ALL due to out-of-memory issues. For individual CRW dataset results without introduced fragmentation, see Supplementary Table S3

Figure  3 shows the alignment error on the 14 CRW datasets without introduced fragmentation. MAFFT(L-INS-i) is not shown because it failed to complete on 16S.3, 16S.T and 16S.B.ALL due to out-of-memory issues (see Section 3.4). Although results depend on individual datasets, certain trends are apparent: MAGUS+eHMMs is the best method with an average error of 10.4%, and PASTA+eHMMs comes as the second with an average error of 11.8%. PASTA is generally less accurate than PASTA+eHMMs and MAGUS+eHMMs (PASTA average error is 12.5%).

### 3.4 Runtime and computational issues

Most analyses were run on the University of Illinois Campus Cluster, and a few large datasets were analyzed on the Blue Waters supercomputer (i.e. high fragmentary 16S.3, 16S.T and 16S.B.ALL, and also the 16S.B.ALL dataset without fragmentation). We set 32 GB memory and 48-hour time limit for all analyses, with 16 CPUs for datasets with fragmentation and 8 CPUs for datasets without introduced fragmentation. All analyses that did not encounter errors completed within the time limit. Some methods had out-of-memory issues and so failed to complete on some datasets; see Supplementary Materials for full details.

Given that the University of Illinois Campus Cluster and the Blue Waters supercomputer consist of heterogeneous computing nodes with different speeds and available memory, it is difficult to draw clear conclusions about runtime for the methods we explore. However, here, we provide some general observations about the wall clock time used in the different analyses; see Supplementary Figures S1, S2, S5, S6 and Supplementary Table S2.

In Experiment 1, our main observation was that using large backbones (all full-length sequences compared with 1000 full-length sequences) and using MAFFT’s techniques for adding fragmentary sequences into backbone alignments increased runtime substantially, making the use of eHMMs to add sequences to 1000-sequence backbone alignments the most efficient technique (Supplementary Figs S1 and S2). In Experiment 2 (Supplementary Fig. S5), we saw that MAGUS+eHMMs was much faster than MAGUS on the HF conditions except for CRW-HF (MAGUS being slightly faster), and somewhat faster on the LF conditions. In Experiment 3 (Supplementary Fig. S6), we compared MAGUS+eHMMs to PASTA, PASTA+eHMMS (i.e. UPP), MAFFT(L-INS-i), MAFFT(default) and MUSCLE. The runtime comparison showed MAFFT(default) to be by far the fastest, especially on large datasets. For example, on the largest dataset, 16S.B.ALL with high fragmentation, MAFFT(default) completed in under an hour while the other methods ranged in time from 6.9 hours (MUSCLE) to 12.9 hours (PASTA). The relative runtimes between methods, after excluding MAFFT(default), do not show any consistent trends, and as noted before, it is not straightforward to interpret these results because of the heterogeneity in the computational environment.

## 4 Discussion

This study revealed differences between methods for alignment of datasets that contain some fragmentary sequences. While the range of model conditions was limited (and in particular, we did not examine any datasets with 30 000 or more sequences nor any protein datasets), we can note the following trends.

We observed that the relative performance between methods depends on the dataset. For example, the differences between methods are reduced on datasets with low average p-distances (indicating low heterogeneity), but are large on the 1000M1 and 1000M2 datasets, which have the highest average p-distances (and so highest heterogeneity). Moreover, with sufficiently low heterogeneity, alignment estimation is generally easy, even when the dataset has fragments, a finding that has been observed in prior studies (Nguyen *et al.*, 2015; Smirnov and Warnow, 2020b).

However, when datasets are challenging to align, then the choice of method can matter substantially. Furthermore, we observed that two-stage methods (i.e. methods that align full-length sequences first and then add the remaining sequences) produce lower alignment error compared with one-stage methods when the level of fragmentation is high, and especially when the rate of evolution is high. Thus, two-stage methods are more robust to fragmentation than one-stage methods. Moreover, of all the methods we tested, MAGUS+eHMMs is the best performing one in terms of alignment accuracy, especially for the hardest model conditions. This is not surprising, since by design two-stage methods enable local alignment strategies (such as the eHMM technique) to be used to add fragmentary sequences into alignments of full-length sequences. More generally, the two-stage approach allows each type of method to be used on those data for which they are primarily designed.

Having said this, it is noteworthy that MAGUS displays fairly good robustness to fragmentation. Thus, with the exception of high levels of fragmentation and high rates of evolution, MAGUS is nearly as accurate as PASTA+eHMMs (i.e. default UPP). MAGUS’ algorithmic design is similar to that of PASTA, and the only important difference is how disjoint alignments are merged. Obviously, this study shows that MAGUS’ technique for merging disjoint alignments (i.e. GCM) is more robust to fragmentation, which is intriguing, and indicates the potential for new method development in alignment merging that could further improve merged alignment accuracy. For example, GCM can be seen as attempting to solve a reformulation of the Maximum Weight Trace problem (Kececioglu, 1993) to the problem of merging disjoint alignments (Zaharias *et al.*, 2021), but other problem formulations may provide even better accuracy.

**Fig. 4.**
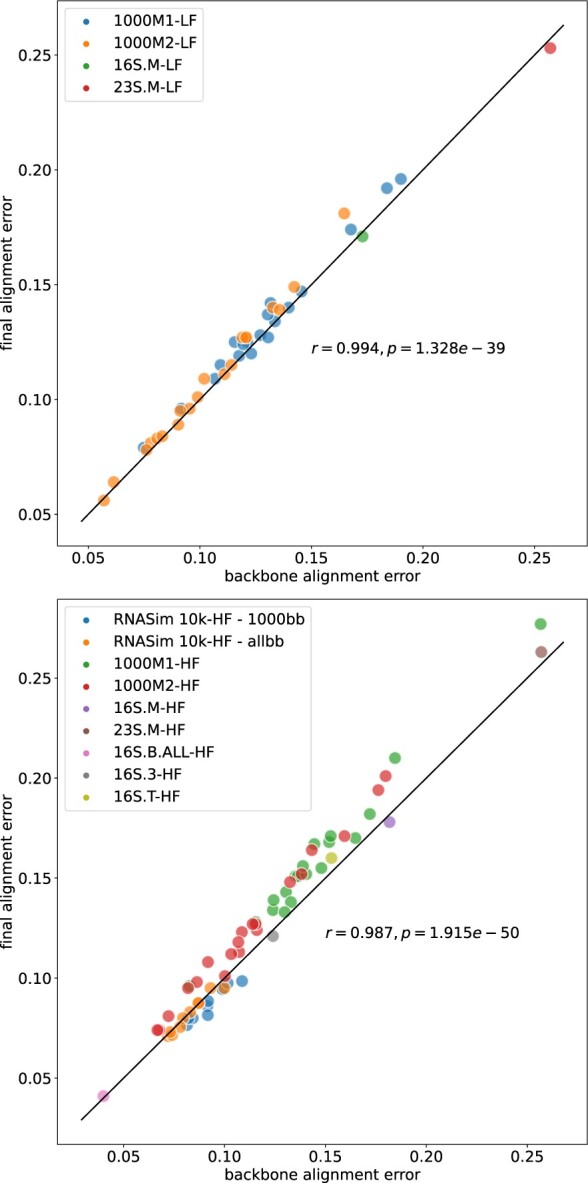
Final versus backbone alignment error for using MAGUS to align the backbone and eHMMs to add the remaining sequences (MAGUS+eHMMs), on low- (top) and high-fragmentation (bottom) conditions. We report the Pearson’s correlation coefficient for each case, showing a strong linear correlation between backbone alignment error and final alignment error across all datasets under both high and low fragmentation

To understand why we see good accuracy in two-stage approaches, we examined the correlation between the backbone alignment error and the final alignment error. Figure  4 shows a very high level of correlation (Pearson’s *r *=* *0.994 and *r *=* *0.987 for low- and high-fragmentation conditions, respectively) between the backbone alignment error in MAGUS+eHMMs and the final alignment error, across all model conditions (results for PASTA+eHMMs show similar trends, see Supplementary Fig. S7). Although the correlation is very high, in general the final alignment error is slightly higher than the backbone alignment error (with bigger increases for the HF conditions than the LF conditions). Furthermore, the points for which the final alignment has noticeably higher error than the backbone alignment are associated with the 1000M1 and 1000M2 datasets on the HF condition, and these are also the points on which we see the highest backbone alignment error. This suggests that the degree to which the eHMM technique maintains the backbone alignment accuracy may be higher for slowly evolving datasets than for quickly evolving datasets, or more generally higher for easy-to-align datasets than for difficult-to-align datasets. Further research is needed to understand whether this (slight) reduction in accuracy can be ameliorated through a more carefully designed sampling strategy than the one we use in this study, which just samples 1000 sequences randomly from the full-length sequences.

## 5 Conclusion

In this study, we examined the problem of computing multiple sequence alignments of datasets that contain fragmentary sequences, a problem that arises in biological sequence analysis in several contexts, and especially when analyzing datasets that contain reads or contigs (such as analyses of metagenomic datasets). The previous leading method for this problem is UPP, which operates by using PASTA to compute an alignment on a sample of the full-length sequences and then adds the remaining sequences into the alignment using an eHMM. We found that replacing PASTA with MAGUS in the UPP pipeline improved accuracy, and some improvements were substantial. Given the noted difficulties in obtaining highly accurate alignments for datasets with fragmentation, MAGUS+eHMMs may provide a valuable addition to the alignment toolkit, with potential benefits to downstream analyses.

We also found that except when the dataset evolves under a high rate of evolution, MAGUS is highly robust to the presence of fragmentation. This property is surprising and may be rare among methods that do not operate in two stages (i.e. by treating full-length sequences separately from fragmentary sequences). Why MAGUS is so robust is not clear, but understanding the algorithmic designs that provide this robustness would benefit developing new methods that are even more robust.

## Funding

This work was supported in part by NSF [2006069, 1458652 to T.W.]. This research is part of the Blue Waters sustained-petascale computing project, which is supported by the US National Science Foundation (awards OCI-0725070 and ACI-1238993) the State of Illinois, and as of December 2019, the National Geospatial-Intelligence Agency. Blue Waters is a joint effort of the University of Illinois at Urbana-Champaign and its National Center for Supercomputing Applications.

*Conflict of Interest*: none declared.

## Data availability

Most of the datasets used in this study are from prior publications. High- and low-fragmentation versions of ROSE 1000M1, 1000M2, CRW 16S.M, 23S.M datasets can be accessed from https://datadryad.org/stash/dataset/doi:10.5061/dryad.95x69p8h8. The three largest CRW datasets (16S.3, 16S.T and 16S.B.ALL) can be accessed from https://sites.google.com/eng.ucsd.edu/datasets/alignment/16s23s. The remaining datasets are available in the Illinois Data Bank at https://doi.org/10.13012/B2IDB-2419626_V1.

## Supplementary Material

btab788_supplementary_data
