# Supplementary material for: MAGUS+eHMMs: improved multiple sequence alignment accuracy for fragmentary sequences
Source: Bioinformatics. 2021 Nov 17;38(4):918–24. doi: 10.1093/bioinformatics/btab788 (PMC8796358; doi:10.1093/bioinformatics/btab788)
Supplement: btab788_supplementary_data [file btab788_supplementary_data.pdf]

S1 Supplementary Materials

Software commands and version numbers

When analyzing datasets with fragmentation including RNASim10k, ROSE 1000M1/M2, CRW 16S.M, 23S.M, 16S.3, 16S.T, 16S.B.ALL, we used 16 CPUs (as denoted below in each command),. For the datasets without fragmentation (i.e., the 14 CRW datasets), we used 8 CPUs. We set 32 GB memory and 48-hour time limit for all benchmarks, and all benchmarks that did not encounter errors finished within the time limit.

1. Command used to record runtime information for each software. The wall clock elapsed time from the output is extracted:

```
$ /usr/bin/time -v <script to run a method>
```

2. Command used to run FastSP (v1.6.0):

```
$ java -ml -mlr -Xmx8192m -jar FastSP.jar \
    -r <reference alignment>  \
    -e <estimated alignment> \
    -o <FastSP output>
```

3. Command used to run PASTA (v1.8.6):

```
$ python3 run_pasta.py \
    -i <unaligned file> \
    -j <output prefix> \
    -d dna --num-cpus=16 \
    -o <output dir>
```

4. Command used to run FastTree (v2.1, multi-threaded version):

```
$ FastTreeMP -nt -gtr <alignment> > <tree>
```

5. Command used to run UPP (v4.5.0, also denoted as PASTA+eHMMs in the paper):

   a) if we have a backbone alignment and tree:

```
$ python3 run_upp.py \
    -x 16 -s <unaligned fragment file> \
    -p <temp dir> -a <backbone alignment> \
    -t <backbone tree> -d <output dir> \
    -o <output prefix>
```

   b) if we do not have a backbone alignment or tree:

```
$ python3 run_upp.py \
    -s <unaligned file> -x 16 \
    -d <output dir> -o <output prefix>
```

6. Command used to run MAGUS. We use MAGUS GitHub version committed on March 9th, 2021. In the default setting, MAGUS is applied recursively on the subsets that contain more than 200 sequences. In our study, we disabled all recursion for MAGUS.

```
$ python3 magus.py -np 16 \
    -i <unaligned file> -d <output dir> \
    -o <output alignment>
    --recurse false
```

7. Commands used to run MAGUS+eHMMs. We modified UPP (v4.5.0) to substitute PASTA by MAGUS to construct the backbone alignment, but the whole pipeline can be easily replicated without modifying UPP. Here we provide detailed instructions on how to run MAGUS+eHMMs without modifying UPP:

   - If we **know** which sequences are full-length and fragmentary, then we randomly select up to 1,000 full-length sequences as backbone sequences. If we **do not explicitly know** which sequences are full-length, then we randomly select up to 1,000 sequences within 25% of the median length (e.g., median length is 1,000 bp, then we randomly select sequences that are 750-1,250 bp).
   - Run Command No.6 on unaligned backbone sequences to obtain a MAGUS backbone alignment.
   - Run Command No.4 on the backbone alignment to obtain a FastTree backbone tree.
   - Run Command No.5a with the backbone alignment ("-a" option), the backbone tree ("-t" option), and the remaining unaligned sequences ("-s" option) as inputs. The final output alignment is the alignment on all sequences.

8. Command used to run MAFFT (default) (v7.475 (2020/Nov/23)):

```
$ mafft --quiet  --thread 16 \
    <unaligned file> > <output alignment>
```

9. Command used to run MAFFT (L-INS-i) (v7.475 (2020/Nov/23)):

```
$ mafft-linsi --quiet  --thread 16 \
    <unaligned file> > <output alignment>
```

10. Command used to run MAFFT to add fragments to an existing backbone alignment (MAFFT(frag)):

```
$ mafft --thread 16 --quiet \
    --addfragments <fragment file> \
    <backbone alignment> > <output alignment>
```

    Using the linsi version to add fragments ("--localpair" option, denoted as MAFFT(linsi-frag)) with "--weighti 0", which optimizes memory usage on possible cost of accuracy. A rough tree of the backbone alignment can be obtained by enabling the "--treeout" option:

```
$ mafft --thread 16 --quiet \
    --localpair --weighti 0 [--treeout] \
    --addfragments <fragment file> \
    <backbone alignment> > <output alignment>
```

11. Command used to run MAFFT to add full-length sequences to an existing backbone alignment using "--add" option [i.e., MAFFT(frag, add)]:

```
$ mafft --thread 16 --quiet \
    --add <unaligned sequence file> \
    <backbone alignment> > <output alignment>
```

    Using the other option "--addfull" [i.e., MAFFT(frag, addfull)]:

```
$ mafft --thread 16 --quiet \
    --addfull <sequence file> \
    <backbone alignment> > <output alignment>
```

12. Command used to calculate Pearson's correlation using Scipy (v1.4.1) with python3:

```
#!/bin/python3
from scipy import stats
r, pvalue = stats.pearsonr( \
    <backbone alignment error>, \
    <final alignment error>)
```

13. Command used to run MUSCLE (v3.8.31). The "-maxiters 2" option is enabled when there are > 3000 sequences in the dataset to align. Notice that MUSCLE does not support multi-processing.

```
$ muscle [-maxiters 2] -in <unaligned file> \
    -out <output path>
```

Additional figures

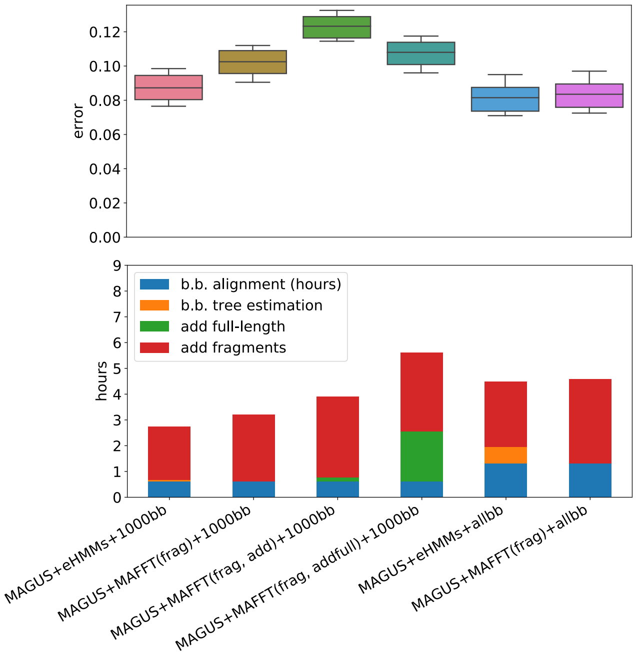

Figure S1: Experiment 1: Average alignment error (average of SPFN and SPFP, top) and average runtime (hours, bottom) analysis for MAGUS backbone conditions on RNASim 10k-HF datasets (10 replicates). The "MAFFT" methods do not require explicit backbone tree estimation. All "MAFFT(frag)" variants add full-length and fragmentary sequences together using "--addfragments". "MAFFT(frag, X)" variants add full-length sequences using strategy "X" and add the fragmentary sequences using "--addfragments". Each method is run on a dedicated node with 16 CPUs, 32 GB memory and a 48-hour runtime limit, and the runtime is taken from the wall-clock elapsed time.

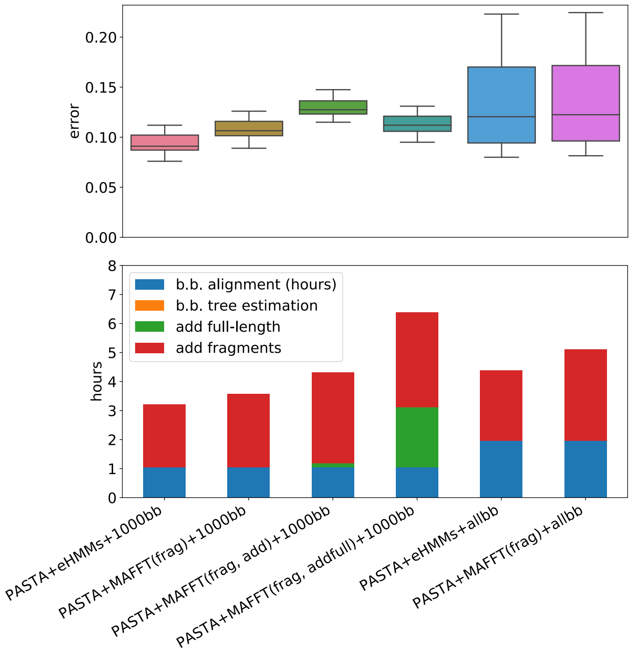

Figure S2: Experiment 1: Average alignment error (average of SPFN and SPFP, top) and average runtime (hours, bottom) analysis for PASTA backbone conditions on RNASim 10k-HF datasets (10 replicates). The "PASTA" variants co-estimate the backbone tree when aligning the backbone sequences. All "MAFFT(frag)" variants add full-length and fragmentary sequences together using "--addfragments". "MAFFT(frag, X)" variants add full-length sequences using strategy "X" and add the fragmentary sequences using "--addfragments". Each method is run on a dedicated node with 16 CPUs, 32 GB memory and a 48-hour runtime limit, and the runtime is taken from the wall-clock elapsed time.

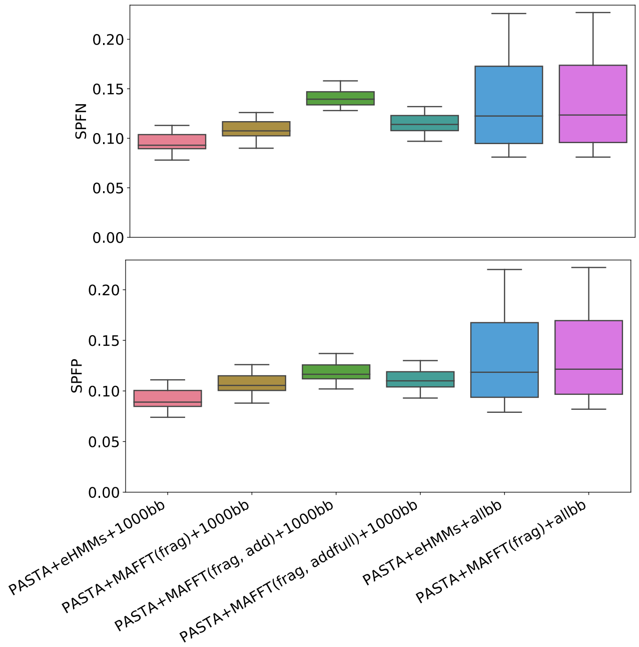

Figure S3: Average SPFN (top) and SPFP (bottom) for all PASTA backbone conditions on RNASim 10k-HF datasets (10 replicates).

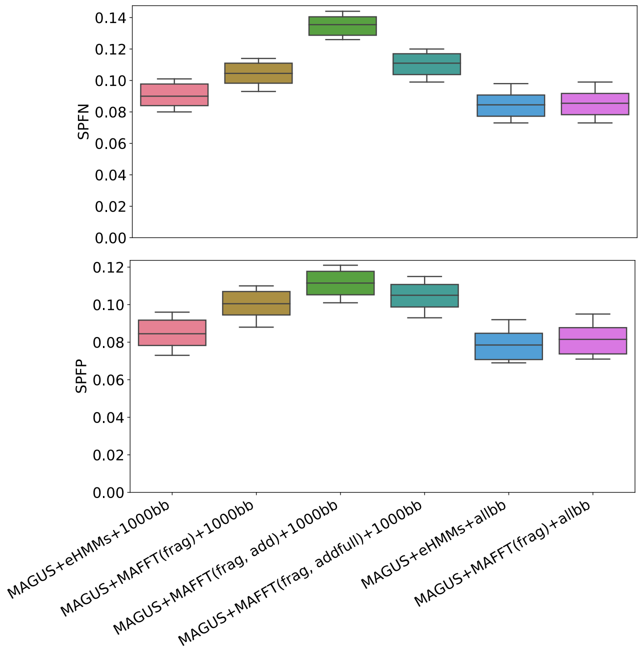

Figure S4: Average SPFN (top) and SPFP (bottom) for all MAGUS backbone conditions on RNASim 10k-HF datasets (10 replicates).

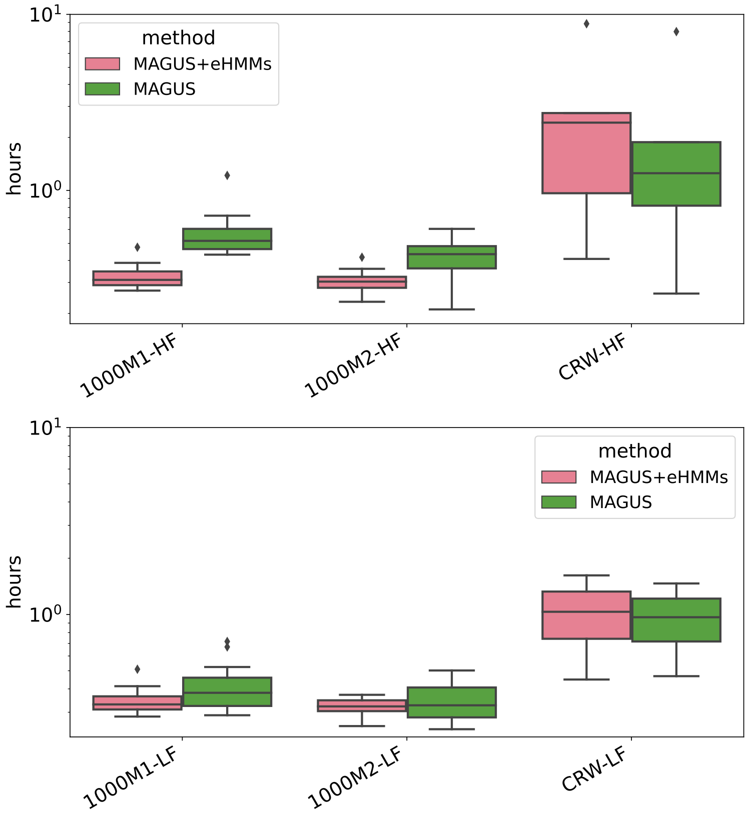

Figure S5: Runtime (hours) in log scale for MAGUS and MAGUS+eHMMs on high (top) and low (bottom) fragmentation conditions. For individual CRW dataset runtime with fragmentation, see Table S2.

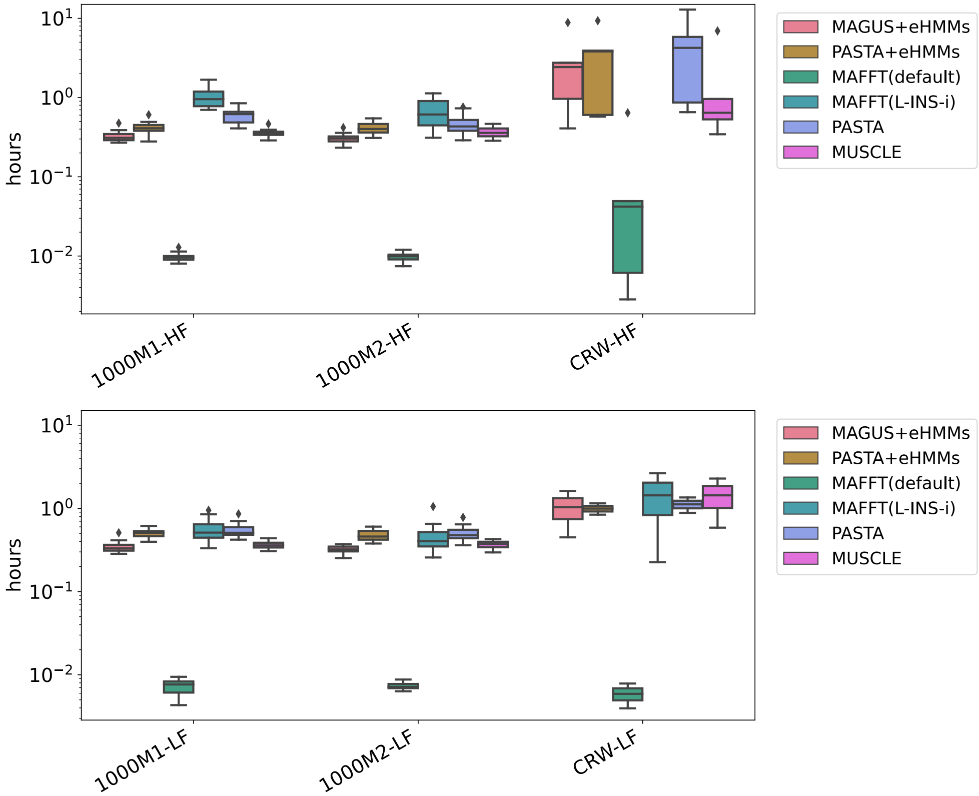

Figure S6: Runtime (hours) in log scale for MAGUS+eHMMs, PASTA+eHMMs, MAFFT(default), MAFFT(L-INS-i), PASTA and MUSCLE on high (top) and low (bottom) fragmentation conditions. For high fragmentary CRW datasets, MAFFT(L-INS-i) failed to complete on one or more datasets due to out-of-memory issues. For individual CRW dataset runtime with fragmentation, see Table S2.

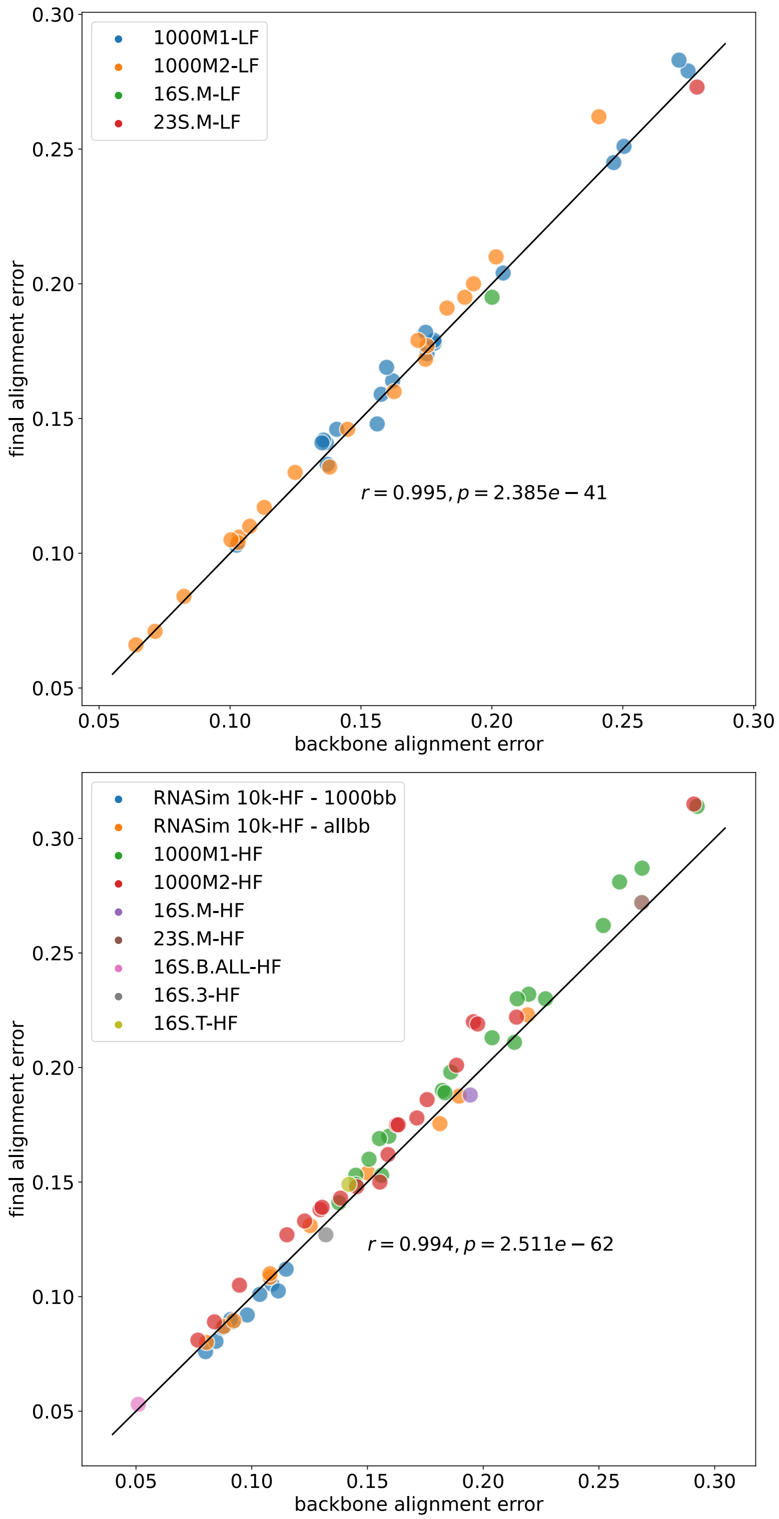

Figure S7: Final versus backbone alignment error for using PASTA to align the backbone and eHMMs to add the remaining sequences (PASTA+eHMMs), on low (top) and high fragmentation (bottom) conditions. The diagonal black line denotes $x = y$, where x is the backbone alignment error and y the final alignment error. We observe that on all datasets, in both high or low fragmentation conditions, there is a strong correlation between the two errors.

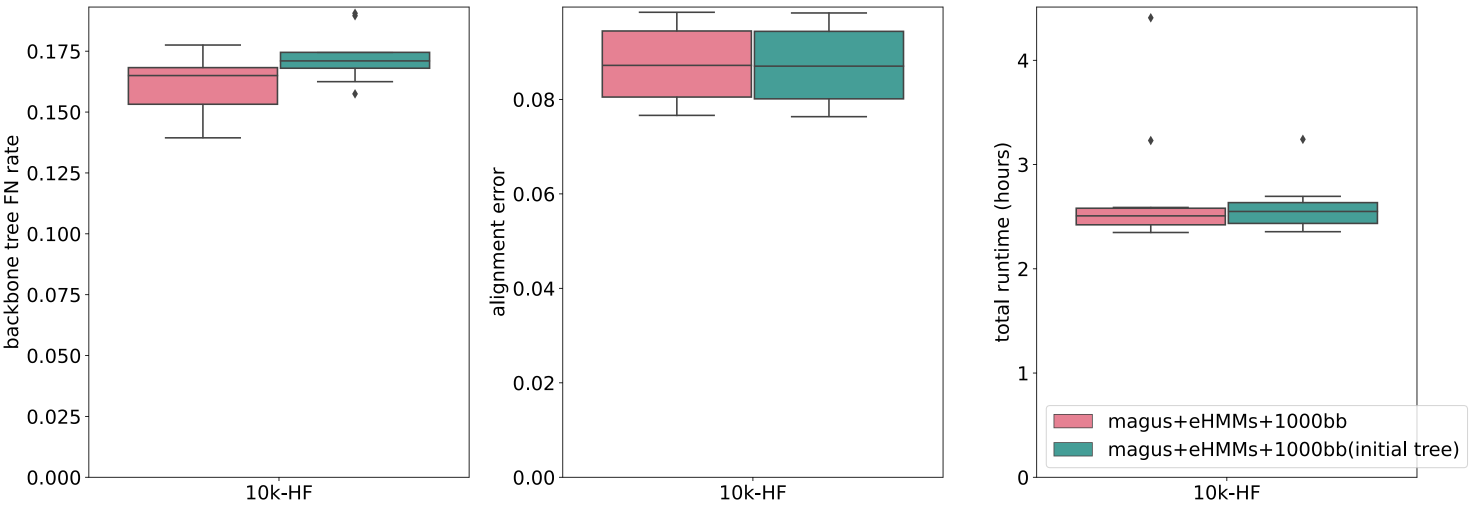

Figure S8: Backbone tree FN rates (left), final alignment error (middle), and total runtime in hours (right) of MAGUS+eHMMs+1000bb (i.e., using MAGUS for backbone alignment on 1,000 full-length sequences, then using the eHMMs technique to add remaining sequences to the backbone alignment) using either a new FastTree tree estimated on the re-estimated backbone alignment (i.e., default MAGUS+eHMMs) or the initial FastTree tree estimated on the initial alignment (denoted with "initial tree"). The dataset is RNASim10k on high fragmentary condition, with 10 replicates. The trees are computed on backbone alignments using FastTree 2 and are fully resolved; thus tree FN and FP rates are the same. See main text Section 3.1 for further discussion.

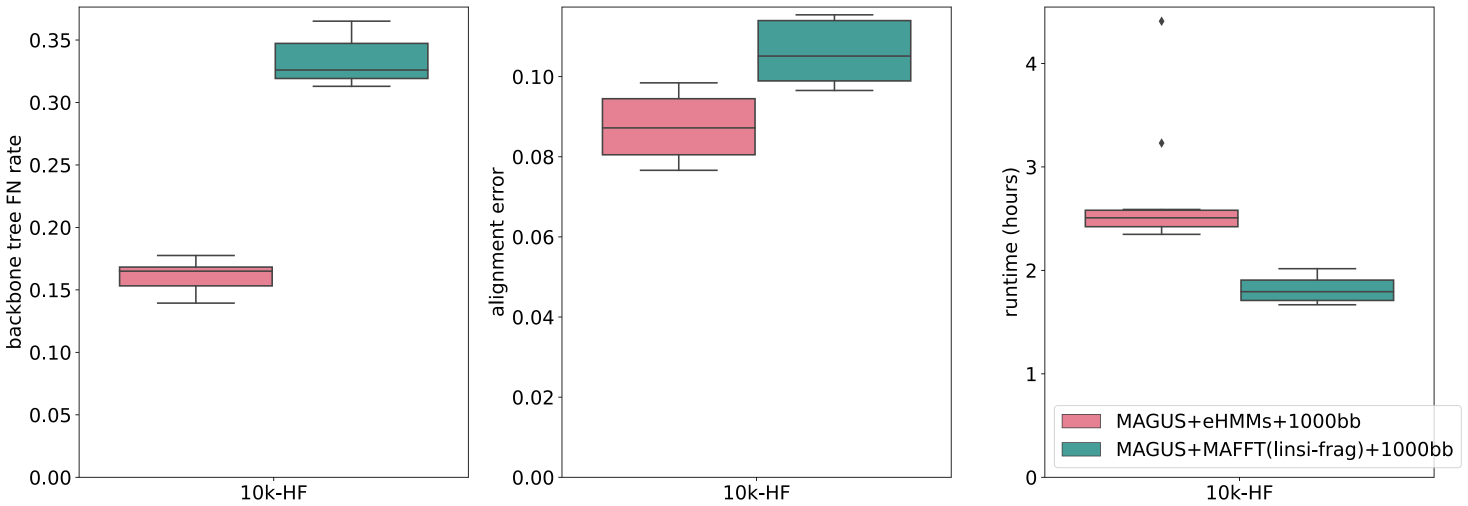

Figure S9: Backbone tree FN rates (left), final alignment error (middle) and total runtime in hours (right) of MAGUS+eHMMs+1000bb (i.e., MAGUS+eHMMs) and MAGUS+MAFFT(linsi-frag)+1000bb. Both pipelines use MAGUS to construct a backbone alignment on 1000 full-length sequences, but the former adds remaining sequences using the eHMMs technique while the latter uses MAFFT(L-INS-I) with "–addfragments" and "–weighti 0" (optimizing memory at the cost of accuracy). The guide (backbone) tree used by MAFFT is obtained by using "–treeout" option. The dataset is RNASim10k on high fragmentary condition, with 10 replicates. The trees are computed on backbone alignments using FastTree 2, and are fully resolved and thus tree FN and FP rates are the same. See main text Section 3.1 for further discussion.

Tables

condition	dataset	MAGUS +eHMMs	MAGUS	PASTA +eHMMs	MAFFT (default)	MAFFT (L-INS-i)	PASTA	MUSCLE
high frag	16S.M	0.167/0.179	0.215/0.192	0.176/0.196	0.230/0.207	0.192/0.200	0.205/0.225	0.403/0.428
	23S.M	0.241/0.290	0.264/0.270	0.242/0.310	0.313/0.343	0.265/0.314	0.327/0.374	0.490/0.531
	16S.3	0.092/0.150	0.083/0.159	0.085/0.169	0.206/0.214	X	0.148/0.172	0.953/0.874
	16S.T	0.136/0.185	0.128/0.187	0.113/0.185	0.275/0.256	X	0.420/0.146	0.969/0.900
	16S.B.ALL	0.043/0.039	0.042/0.036	0.054/0.052	0.162/0.092	X	0.092/0.075	0.976/0.911
	average error	0.152	0.157	0.158	0.230	-	0.218	0.743
	median error	0.160	0.157	0.149	0.219	-	0.215	0.913
low frag	16S.M	0.165/0.177	0.276/0.170	0.182/0.202	0.233/0.209	0.175/0.184	0.396/0.229	0.394/0.345
	23S.M	0.222/0.282	0.223/0.278	0.239/0.311	0.301/0.320	0.242/0.303	0.239/0.314	0.395/0.438
	average error	0.212	0.237	0.234	0.266	0.227	0.294	0.393
	median error	0.212	0.237	0.234	0.266	0.227	0.294	0.393

Table S1: SPFN/SPFP for individual CRW datasets with high and low fragmentation conditions. The average/median errors are measured using alignment errors (average of SPFN and SPFP). Each method is run on a dedicated node with 16 CPUs, 32 GB memory and a 48-hour runtime limit. "X" means that the method did not complete within the time limit or encountered out-of-memory issues for that dataset.

condition	dataset	MAGUS +eHMMs	MAGUS	PASTA +eHMMs	MAFFT (default)	MAFFT (L-INS-i)	PASTA	MUSCLE
high frag	16S.M	0.408	0.259	0.575	0.003	1.178	0.654	0.959
	23S.M	0.964	0.819	0.604	0.006	0.173	0.866	0.344
	16S.3	2.426	1.252	3.821	0.042	X	4.237	0.530
	16S.T	2.751	1.880	3.912	0.049	X	5.828	0.643
	16S.B.ALL	8.844	7.979	9.325	0.642	X	12.892	6.936
	average runtime (hours)	3.078	2.438	3.647	0.148	0.676	4.895	1.882
low frag	16S.M	0.449	0.468	0.841	0.004	2.644	0.884	2.285
	23S.M	1.619	1.466	1.149	0.008	0.226	1.354	0.586
	average runtime (hours)	1.034	0.967	0.995	0.006	1.435	1.119	1.436

Table S2: Runtime in hours for individual CRW datasets with high and low fragmentation conditions. Each method is run on a dedicated node with 16 CPUs, 32 GB memory and a 48-hour runtime limit. "X" means that the method did not complete within the time limit or encountered out-of-memory issues for that dataset.

	MAGUS +eHMMs	MAGUS	PASTA +eHMMs	MAFFT (default)	MAFFT (L-INS-i)	PASTA	MUSCLE
5S.E	0.031/0.014	0.028/0.026	0.021/0.016	0.094/0.069	0.019/0.019	0.019/0.020	0.169/0.136
5S.3	0.087/0.086	0.099/0.104	0.102/0.101	0.245/0.186	0.158/0.158	0.152/0.156	0.423/0.320
5S.T	0.116/0.116	0.092/0.099	0.186/0.186	0.235/0.180	0.131/0.131	0.219/0.218	0.485/0.371
16S.A	0.021/0.023	0.023/0.024	0.023/0.024	0.030/0.027	0.023/0.024	0.024/0.026	0.046/0.039
16S.B.ALL	0.045/0.044	0.044/0.045	0.054/0.054	0.176/0.100	X	0.066/0.064	0.428/0.245
16S.C	0.039/0.044	0.039/0.043	0.039/0.045	0.048/0.045	0.038/0.043	0.040/0.046	0.068/0.069
16S.M	0.201/0.174	0.179/0.179	0.218/0.241	0.249/0.215	0.184/0.195	0.217/0.241	0.317/0.290
16S.3	0.079/0.148	0.088/0.153	0.091/0.181	0.243/0.240	X	0.089/0.174	0.272/0.243
16S.T	0.129/0.176	0.128/0.167	0.112/0.190	0.295/0.269	X	0.124/0.202	0.350/0.306
23S.A	0.074/0.075	0.075/0.076	0.077/0.081	0.078/0.078	0.070/0.076	0.095/0.094	0.092/0.093
23S.C	0.036/0.043	0.042/0.045	0.035/0.043	0.055/0.053	0.038/0.046	0.034/0.042	0.066/0.067
23S.E	0.177/0.204	0.177/0.204	0.175/0.212	0.202/0.213	0.177/0.208	0.179/0.215	0.210/0.232
23S.M	0.214/0.268	0.226/0.262	0.229/0.301	0.298/0.308	0.231/0.286	0.246/0.318	0.361/0.406
23S.3	0.116/0.123	0.109/0.116	0.114/0.137	0.151/0.140	0.107/0.120	0.113/0.138	0.211/0.196
average error	0.104	0.103	0.117	0.161	-	0.128	0.233
median error	0.100	0.098	0.114	0.176	-	0.128	0.239

Table S3: SPFN/SPFP of tested methods on each CRW biological dataset without introduced fragmentation, including MAFFT(L-INS-i) results. The average/median errors are measured using alignment errors (average of SPFN and SPFP) over all 14 datasets. Therefore, MAFFT(L-INS-i) is excluded as it fails to complete on three datasets. Each method is run on a dedicated node with 8 CPUs, 32 GB memory and a 48-hour runtime limit. "X" means that the method did not complete within the time limit or encountered out-of-memory issues for that dataset.

condition	dataset	MAGUS +eHMMs	MAGUS	PASTA +eHMMs	MAFFT (default)	MAFFT (L-INS-i)	PASTA	MUSCLE
high frag	1000M1(19)	0.177/0.141	0.322/0.252	0.227/0.187	0.997/0.994	0.665/0.509	0.629/0.608	0.805/0.820
	1000M2(20)	0.140/0.111	0.210/0.187	0.180/0.151	0.995/0.990	0.531/0.395	0.446/0.430	0.777/0.791
low frag	1000M1(19)	0.143/0.123	0.154/0.138	0.188/0.172	0.997/0.992	0.435/0.304	0.223/0.214	0.669/0.668
	1000M2(20)	0.115/0.100	0.116/0.107	0.151/0.141	0.993/0.984	0.334/0.226	0.170/0.165	0.640/0.638

Table S4: average SPFN/SPFP for simulated datasets (1000M1 and 1000M2, numbers of replicates are marked next to dataset names) with high and low fragmentation conditions. Each method is run on a dedicated node with 16 CPUs, 32 GB memory and a 48-hour runtime limit.

MAFFT(L-INS-i) out-of-memory issue on adding many sequences

When trying to use MAFFT L-INS-i to add fragmentary sequences into the 1000-sequence backbone alignments (using a dedicated node with 16 CPUs, 32 GB memory, and a 48-hour time limit) on the RNASim 10K-HF datasets, we received the following out-of-memory error from SLURM:

```
$ start at 2021-04-14 17:59:43

$ thread: 16, method: magus+mafftadd+1000bb
$ full-length file: /home/chengze5/tallis/download/10k-HF/R1/unaligned-full-1000bb.fas
$ frag file: /home/chengze5/tallis/download/10k-HF/R1/unaligned-frags-1000bb.fas
$ end at 2021-04-14 21:08:43

$ slurmstepd: error: Detected 1 oom-kill event(s) in StepId=2048431.batch cgroup. Some of your
    processes may have been killed by the cgroup out-of-memory handler.
```

We received a similar error from SLURM when trying to add sequences to an "allbb" backbone.

```
$ start at 2021-04-14 17:59:43

$ thread: 16, method: magus+mafftadd+allbb
$ full-length file: /home/chengze5/tallis/download/10k-HF/R1/unaligned-full.fas
$ frag file: /home/chengze5/tallis/download/10k-HF/R1/unaligned-frags.fas
$ end at 2021-04-15 00:35:05

$ slurmstepd: error: Detected 1 oom-kill event(s) in StepId=2048432.batch cgroup. Some of your
    processes may have been killed by the cgroup out-of-memory handler.
```
